# Drowning and Nonfatal Drowning in Children and Adolescents: A Subsequent Retrospective Data Analysis

**DOI:** 10.3390/children11040439

**Published:** 2024-04-06

**Authors:** Sebastian Berger, Manuela Siekmeyer, Stefanie Petzold-Quinque, Wieland Kiess, Andreas Merkenschlager

**Affiliations:** 1 Department of Women and Child Health, Hospital for Children and Adolescents, Division of Neuropediatrics, University of Leipzig, Liebigstraße 20a, 04103 Leipzig, Germany; andreas.merkenschlager@medizin.uni-leipzig.de; 2 Department of Women and Child Health, Hospital for Children and Adolescents, Pediatric Intensive Care Unit, University of Leipzig, Liebigstraße 20a, 04103 Leipzig, Germany; 3 Department of Women and Child Health, Hospital for Children and Adolescents and Center for Pediatric Research, University Hospital Leipzig, Liebigstraße 20a, 04103 Leipzig, Germany

**Keywords:** drowning prevention, submersion, children and adolescents, neurological outcomes, prognostic factors

## Abstract

Fatal and nonfatal drowning are among the leading causes of death and lifelong severe neurological impairment among children and adolescents. This study aimed to complement research from Leipzig 1994–2008 to seek trends within risk factors, treatments, and outcomes throughout the last decade. We retrospectively investigated data of 47 inpatients aged 0–18 admitted to Leipzig University Department of Pediatrics who matched ICD-10 code T75.1 from 2008 to 2020 and compared them to a preceding study at the same institution. We also examined the prognostic value of parameters regarding the patients’ outcomes. There were three median incidents per annum. The median age was 2.75 years; 76% of incidents happened in males. An accumulation was seen during the summer months and weekends. Most drowning incidents occurred in private ponds or pools (48.9%). Thirty-nine children were discharged without resulting morbidity, four showed neurological impairment, and three died. Risk factors concerning age, sex, and incident characteristics were confirmed. Special supervision needs still apply to 1–3-year-old male children or children with pre-existing health conditions around private pools and ponds. Hospitalization duration shortened, and morbidity and lethality decreased since the previous study. There was structural improvement in primary care and medical documentation. Parameters suggesting good outcomes include a submersion time < 5 min, GCS > 3 points, spontaneous movement upon admission, remaining pupillary light response, the absence of cardiovascular arrest, body temperature ≥ 32 °C, pH > 7, blood glucose < 15 mmol/L, lactate < 14 mmol/L, base excess ≥ −15 mmol/L, and the absence of ARDS. Clear legislation can contribute to improved private home water safety. Further studies should include a broad in- and outpatient spectrum and standardized incident documentation presupposing Utstein-style reporting. Regular reinvestigation of consistent geographical regions facilitates process evaluations of drowning epidemiology and therapy evolution.

## 1. Introduction

In every part of the world, drowning is among the ten leading causes of death among children and adolescents [1]. In Germany, 47 children from ages 0 to 20 fell victim to fatal drowning accidents in 2021 [2], thus constituting the second most common cause of accidental death in children and adolescents [3]. The number of nonfatal drownings exceeds this by multiple times [4].

Drowning is understood as “the process of experiencing respiratory impairment from submersion/immersion in liquid” [5,6]. This process begins when a person’s airway submerges below a fluid’s surface, called submersion, or when a liquid suffuses the person’s respiratory tract, called immersion. As a result, water enters the mouth or nose and is willingly spewed out or swallowed. Either the failure to consciously hold one’s breath or further resist the respiratory drive leads to the aspiration of liquid into the respiratory system [7].

An incident leading to death at the scene or within 24 h following the incident is referred to as a fatal drowning [8]. If rescue results in the victim’s survival, this is called nonfatal drowning. Water-related submersion accidents without an aspiration are cited as water rescues [7,9]. The precise physiological process of drowning is intricate [7]. Water entering the mouth or nose may lead to aspiration and mechanical injury to the glottis and lung tissue. Both hyper- and hypotonic liquids provoke alterations in the pulmonary surfactant, impairing gas exchange processes [7]. Systemic blood oxygen deficiency results in unconsciousness and further apnea [10]. 

The main cause of death after drowning accidents is due to hypoxic damage to the central nervous system [11]. Secondary damage includes hypoxia-associated consequential organ failure, rhabdomyolysis, disseminated intravascular coagulation, and acute renal failure [10,12]. Of the children who survive nonfatal drowning, many suffer from long-term consequences such as neurological impairment, like motor and cognitive disorders, e.g., spasticity and dystonia or vegetative states of consciousness [5,10,13].

However, drowning is preventable [1,9,10]. Knowledge about risk factors has already resulted in a recent decrease in fatal drowning accidents in high-income countries throughout the world [4,5,14]. For about three decades, studies have aimed to investigate incident characteristics and predictive parameters to devise instructive guidelines [10,11,15,16,17,18,19]. There are few proven risk factors or reliable data concerning children’s clinical course or outcome-related prognostic features [5].

In order to specify the research questions of this work, we briefly refer to the current state of research and outline our motivation for further investigation.

### 1.1. Epidemiology and Prevention

According to Girasek et al., fatal drowning has evolved from the second most common to leading cause of death for children aged 1–4 years in the US within the last decade [20]. Fatal-drowning-related mortality rates in US hospitals range from 10 to 17% [21]. Drowning rates have been found to have decreased from 2010 to 2019, with a significant increase in 2020, as reported by Moreland et al., possibly linked to societal changes due to the coronavirus disease [22]. 

Fatal drowning is a predominant issue in low- and middle-income countries, as estimated by Franklin et al. in 2017 [23]. However, the full extent of drowning is underreported worldwide [24] according to Meddings, resulting in an understudied research topic.

Internationally confirmed and repeatedly cited risk factors are male sex—fatal drowning rates among males are at least twice the rates in females—and very young age [1,20,22]; pre-existing health conditions like epilepsy, cardiac arrhythmias, notably long-QT syndrome, or patients with autism spectrum disorder [25]; swimming pools for young children and natural bodies of water for adolescents [26]; and the simultaneous consumption of alcohol, revealed in a review by Driscoll et al. [27].

Reviews analyzing approaches to drowning prevention have found effective measures to be continuous one-on-one supervision [28,29]; professional daycare [30]; physical boundaries around bodies of water [31]; educational water competency training for children older than 1 year and caregivers [28,32]; and the use of personal flotation devices [1,14]. It should also be highlighted that research gaps can be addressed by regular and continuous re-evaluation, as circumstances may alter over the course of time [20,24].

Our aim was to see whether data from our geographic region reflect international trends and would add to these solid research assumptions while at the same time conducting a status update of our location.

### 1.2. Management and Interventions 

First and foremost, drowning management requires the safe removal of affected persons from the water and applying basic cardiopulmonary resuscitation (CPR) if required, mostly by non-medical bystanders, so adequate education of the population is required [33,34]. All drowning victims should undergo fast checking for cardiac activity and hypothermia so that therapy efforts can be quickly initiated. Ventilatory assistance may be necessary, as it has been associated with better neurologic outcomes and higher survival rates [33].

At the emergency department, Bierens et al. recommend extensive evaluations of the circulatory status and organ function (blood pressure, respiratory rate, pulse oximetry, capnography, chest X-ray, blood glucose, creatinine, and electrolytes) and thereby appointing adequate treatment measures, perhaps including endotracheal intubation [20,35]. Recent research has proposed that direct discharge may be safe from the emergency department after 6 h when there is no mentation deficit, no need for supplemental oxygen, and consistently unimpaired vital signs [33]. 

In contrast to the comprehensive data on prevention, many authors conclude that there is little evidence regarding drowning first responses and effective clinical care pathways [33,35]. There is a lack of randomized controlled trials [36], especially on the issues of “resuscitation, airway management, oxygen administration, use of automated external defibrillator, bystander CPR, ventilation strategies, extracorporeal membrane oxygenation, and […] hospital discharge” [33]. Szpilman and Morgen denounced a “deficit of high-quality scientific evidence at all stages of the patient’s journey following a drowning event, particularly in the hospital” [35]. Researchers call for the application of Utstein-style documentation to enable research leading to evidence-based guidelines [16,33,37].

We found it insightful to describe the clinical care our patient collective underwent and what measures were applied in terms of emergency responses.

### 1.3. Prognosis

Most children survive drowning incidents without any implications [20]. However, in a review from 2012, Suominen et al. described the severity of possible drastic neurologic outcomes following nonfatal drowning [38], namely, lifelong neurologic impairment, backed by other authors investigating the impact of fatal and nonfatal drowning [11,13,19].

It is a clinician’s ideal to draw prognostic assumptions at an early stage so as to conclude the appropriateness of further treatment steps. Promising variables for the prognostication of outcomes are submersion time; primary neurological (GCS) and cardiopulmonary function (pulse; blood pressure); body temperature; laboratory tests like pH, blood sugar, and lactate levels; plain chest X-ray results; and pre-existing health-conditions [10,11,15,16,17,18,19,38,39]. 

An often-cited predictor of drowning outcomes is submersion time [37], with some studies discussing submersion > 25 min [40] and some quoting submersion > 10 min as presaging a fatal outcome [6,18,41]. Young age is acknowledged to be associated with a better prognosis [37], while male sex might be associated with fatal outcomes [10,42,43]. 

A review from 2020 notes inconsistency in the application of definite outcome parameters due to yet missing analyses distinguishing significant variables from confounding factors [37].

As a recent “state-of-the-art review” predicts a growing number of deadly drownings in the following years due to climate change [44], we aimed to fill in research gaps and investigate whether the refinement of medical and rescue techniques has perhaps, contrariwise, led to improved figures in mortality and morbidity in our geographical region.

From 2008 to 2020, 47 children were admitted to Leipzig University Department of Pediatrics (LDP) due to drowning incidents. We aim to provide an overview of recurring risk factors and assess prognostic assumptions to reduce incident frequency and enable more precise clinical evaluations of affected patients.

## 2. Materials and Methods

### 2.1. Participants 

Retrospective data analysis included all inpatients treated at Leipzig University Department of Pediatrics (LDP) with the ICD-10 discharge diagnosis T75.1 (drowning and nonfatal submersion) from July 2008 to December 2020. As a university hospital, LDP covers not only the city of Leipzig but also surrounding communities and, partially, other federal states, as it holds a highly equipped pediatric intensive care unit.

The named criteria applied to 47 patients aged 0–18 years. For one patient (2.1%), the overall outcome could not be obtained from their medical file. This patient was included in the epidemiologic data description but excluded from the assessment of patient outcomes and referring parameters.

The children’s ages ranged from 6 months (minimum 0.5 years) to 17 years (maximum, 17.083 years), with a mean age of 4.703 years (SD ± 4.42 years). The median was 2.75 years. There were more incidents in males (*n* = 36 (76.6%)) than in females (*n* = 11 (23.4%)). The children were also analyzed regarding existing comorbidities prior to the drowning incident. See Supplementary Materials.

### 2.2. Procedure

We used “Data Warehouse” (electronic hospital database SAP SE, Walldorf, Germany, Version EHP8 FOR SAP ERP 6.0 SPS12) to identify an eligible patient collective. Data were obtained from patients’ digital medical records and the pediatric intensive care unit (PICU) patient data management system, “COPRA” (PDMS COPRA System GmbH, Berlin, Germany).

### 2.3. Measures 

We utilized a questionnaire in accordance with that of Brüning [11] consisting of 88 items of the following categories: (1) patient and accident characteristics, (2) parental situation, (3) first aid, (4) clinical presentation upon admission, (5) diagnostic/laboratory tests upon admission, (6) clinical course, and (7) patient’s overall outcome. Outcome was dichotomized into (a) no resulting morbidity and (b) neurological impairment or death. Results are presented in a narrative way.

### 2.4. Data Analysis 

The data set was categorized in “Microsoft Excel” (Microsoft Corporation, Version 16.7) and analyzed using the IBM software “Statistical Package for the Social Sciences” (SPSS Statistics, IBM, Armonk, NY, USA, Version 29). The raw data file can be downloaded at: https://www.mdpi.com/article/10.3390/children11040439/s1, Table S1: RAW_DATA_FILE_ DROWNING.xlsx. Besides descriptive interpretation, we examined the prognostic value of parameters on the patients’ overall outcomes by applying cut-offs and relating them to the outcome groups through cross-tables. Results were assessed for significance via Mann–Whitney test. Relative risk (RR) and 95% confidence intervals (95%CI) were calculated and assessed using the χ^2^ test. We supposed *p* < 0.05 as the hallmark of a significant result. To seek trends since the year 2008, we performed a comparison with data from Brüning et al. [11]. Their study was also carried out at LDP and focused on similar aspects of drowning incidents.

## 3. Results

### 3.1. Patient Characteristics

**Figure 1:**
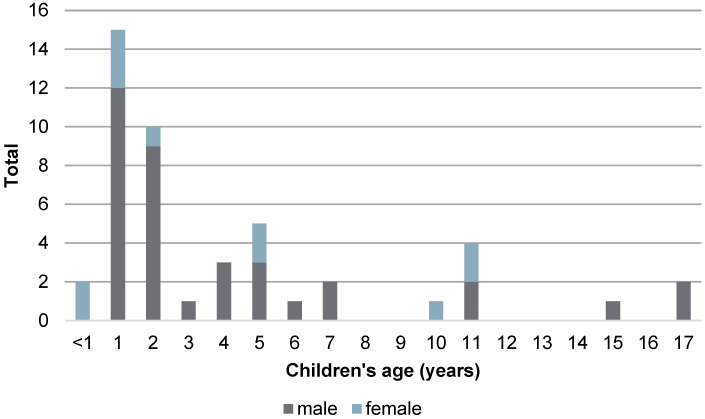
Age distribution (47 children).

In total, 47 children qualified for the survey. Six children (12.8%) had some form of a pre-existing condition (chronic or acute disease) prior to the incident, e.g., dyskinetic cerebral palsy (*n* = 1), symptomatic focal or generalized epilepsy (*n* = 3), psychomotor retardation (*n* = 2), Dandy–Walker malformation (*n* = 1), and intellectual disability of unknown origin (*n* = 1). One patient had been suffering from an infection of the upper respiratory tract for three days, and one had experienced a falling accident 6 months earlier. While most children’s parents were either married or in a permanent relationship (*n* = 35 (74.5%)), a quarter of the patients had single or separated parents (*n* = 12 (25.5%)). For age distribution, see Figure 1.

### 3.2. Accident Features

**Figure 2:**
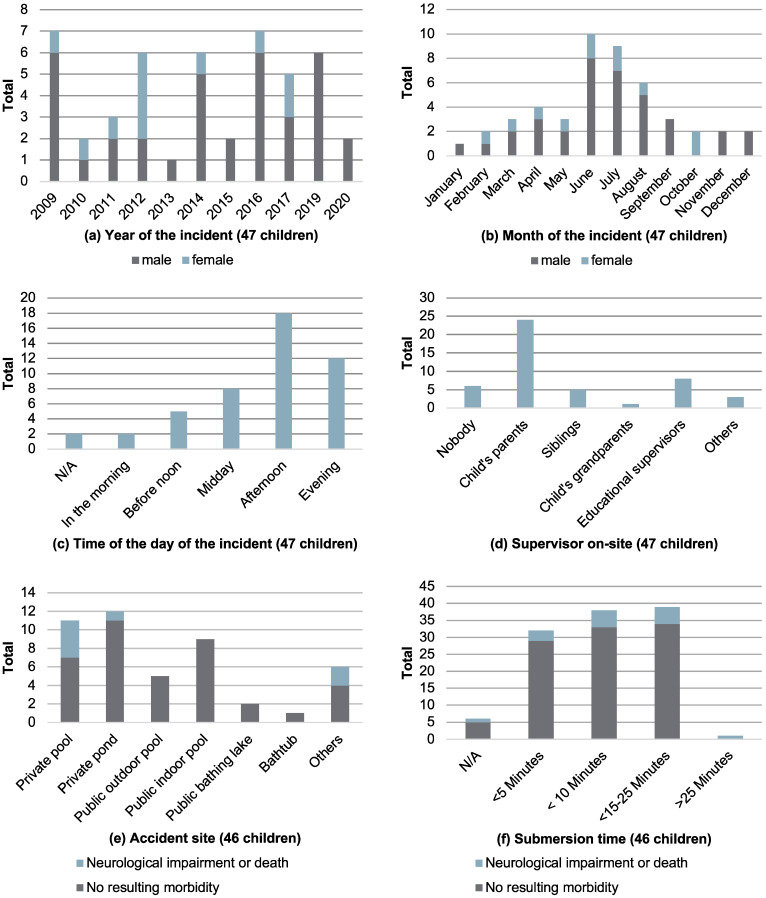
Accident features (**a**–**f**).

In the median, there were three drowning incidents per annum (minimum, 0; maximum, 7; mean, 3.615; median, 3; SD ± 2.631). An accumulation during the weekend was seen (*n* = 20 (42.6%)). The majority of accidents took place in the afternoon, from 2 pm to 6 pm (*n* = 18 (38.3%)), followed by the evening hours, 6 pm to 10 pm (*n* = 12 (25.5%)). Half the children were with their parents (*n* = 24 (51.1%)) or educational supervisors (*n* = 8 (17.0%)), while most of the cases occurred in private ponds (*n* = 12 (25.5%)) or pools (*n* = 11 (23.4%)). There was also a drowning incident in a bathtub (*n* = 1 (2.1%)). Distributions over the years, months, supervising instances, and accident sites are depicted in Figure 2. Estimated submersion times lasted from less than a minute (*n* = 16 (34.0%)) up to more than 25 minutes (*n* = 1 (2.1%)). In most cases, a submersion time range of 1 to <5 min was reported (*n* = 17 (36.2%)). For a graphical representation of submersion times (Figure 2f), we subcategorized in accordance with the current relevant literature [40] (see Figure 2a–f).

### 3.3. Primary Care

In 31 cases (66.0%), an ambulance was deployed, of which 27 (87.1%) were accompanied by an emergency physician. The mean time until arrival at the incident site was just under ten minutes (minimum, 3.00 min; maximum, 24.00 min; mean, 9.926 min; median, 10.00 min; SD ± 4.875 min). Ten patients (21.3%) were admitted to the hospital by rescue helicopter. In 24 children (51.1%), cardiopulmonary resuscitation (CPR) was performed by non-medical bystanders. In seven patients (14.9%), CPR was initially or additionally carried out by the emergency physician on-site. The median duration of reanimation amounted to 25.00 min (minimum, 1.00 min; maximum, 180.00 min; mean, 39.000 min; SD ± 54.328 min). A quarter were intubated prior to hospital admission (*n* = 12 (25.5%)). Eight patients (17.0%) received adrenaline or noradrenaline. In seven cases (14.9%), they were administered other medications for cardiovascular support, i.e., dobutamine and atropine.

Ten patients (21.3%) showed seizures, and half of these (*n* = 4 (8.5%)) received antiseizure medication.

### 3.4. Hospital Admission

**Figure 3:**
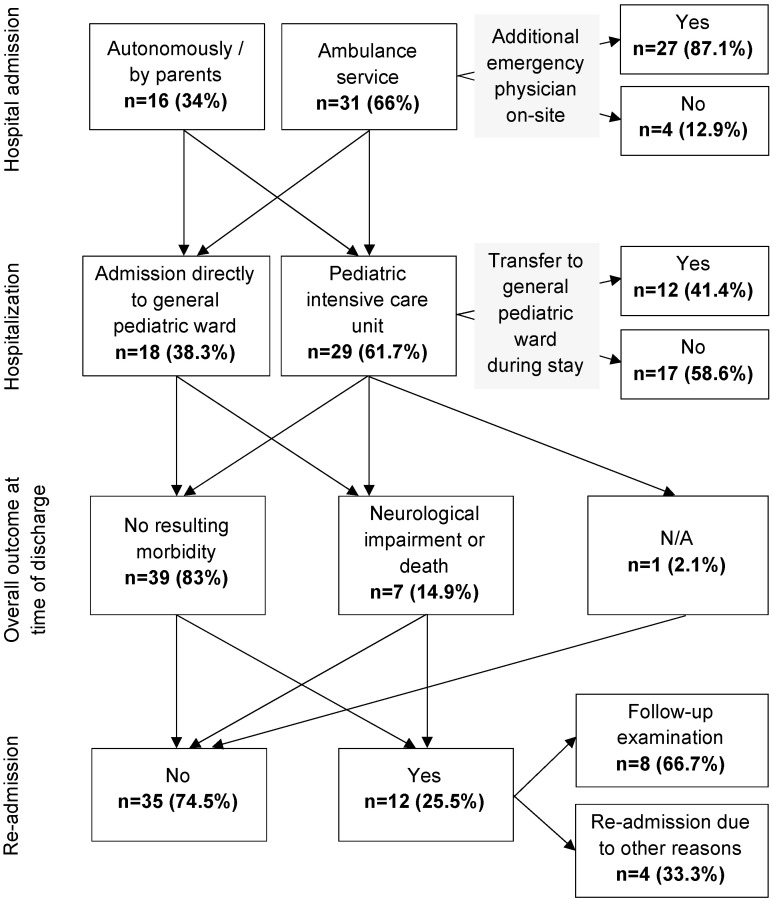
Clinical course.

An overview of the patients’ clinical courses can be retraced in Figure 3. The patients were either transported to Leipzig University Department of Pediatrics (LDP) by medical ambulance service or presented themselves autonomously and with their caregivers. According to their clinical presentations, they were admitted directly to the general pediatric ward or the pediatric intensive care unit (PICU). In some cases, children were later transferred from the PICU to the general pediatric ward, from where they could be discharged or sent to rehabilitation.

**Figure 4:**
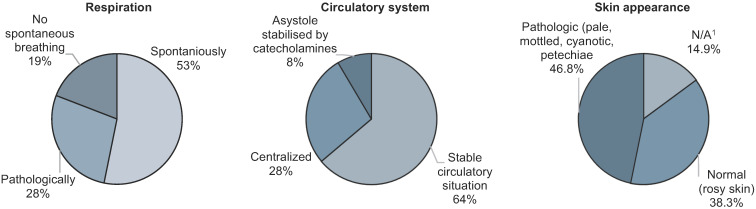
Cardiopulmonary situation upon admission (47 children). ^1^ Not applicable.

The children’s cardiopulmonary statuses upon arrival were documented as shown in Figure 4. While more than half the patients (*n* = 25 (53.2%)) were breathing spontaneously, 13 (27.7%) showed pathological breathing patterns, and a fifth (*n* = 9 (19.1%)) showed no spontaneous respiration at all. Pulmonary auscultation findings were pathological in 18 children (38.3%); conversely, 26 (55.3%) had a physiological pulmonary auscultation result. Rosy skin could be observed in 18 children (38.3%), whereas, in 22 cases (46.8%), the skin showed a pathologic appearance, e.g., pale. mottled, cyanotic, or visible petechiae. In seven children (14.9%), no visual impression was documented. On average, the children arrived at the hospital with a body temperature of 34.973 °C, and the median was 36.500 °C (minimum, 27.0 °C; maximum, 38.4 °C; SD ± 3.079 °C). Systolic blood pressure ranged from 45 mmHg to 160 mmHg (mean, 110.343 mmHg; median, 110 mmHg; SD ± 20.253 mmHg), and diastolic blood pressure ranged from 20 mmHg to 105 mmHg (mean, 63.314 mmHg; median, 60 mmHg; SD ± 17.008 mmHg). The children’s median heart rate upon arrival was 112 bpm, with a minimum of 65 bpm and a maximum of 150 bpm (mean, 113.262 bpm; SD ± 21.112 bpm). The median oxygen saturation (SpO_2_) was 96%.

For selected laboratory testing results, see Table 1. In terms of blood pH, the median pH observed was acidotic at 7.305, while the base excess showed a median of −7.832 mmol/L. Blood lactate amounted to a median of 3.6 mmol/L. The neuron-specific enolase (NSE) was constantly elevated at a median of 49.40 µg/L (mean, 51.571 µg/L; SD ± 14.284). Median and average blood glucose levels were above the norm, and the median leucocyte count of 11.8 × 10^9^/L remained just within the range of physiological references.

Clinical testing results upon hospital admission can be found in Table 2. Three-quarters (*n* = 36 (76.6%)) were moving spontaneously. In total, 45 patients (95.7%) showed pupils that were round and equal in size; in two children (4.3%), they were distorted. Most children still had a prompt response to light (*n* = 38 (80.9%)), with three (6.4%) delayed; in four cases (8.5%), there was none. The Glasgow Coma Scale (GCS), performed on 34 patients (72.3%), showed a result of 3 points in 10 patients (21.3%) and above 4 in 24 patients (51.1%).

### 3.5. Hospitalization

The number of days in the PICU was one in the median (minimum, 0 d; maximum, 40 d; mean, 3.565 d; median, 1.000 d; SD ±7.626 d). In the general pediatric ward, patients remained for 1.5 days on average (minimum, 0 d; maximum, 8 d; mean, 1.511 d; median, 1.000 d; SD ± 2.02 d). 

The children showed different treatment needs depending on the severity of their clinical impairment. In total, 14 children (29.8%) were intubated within the clinic, and 17 (36.2%) received respiratory support, e.g., oxygen nasal cannulae. Intubation was maintained for up to nine days (minimum, 0 h; maximum, 216 h; mean, 21.033 h; SD ± 47.335 h), and respiratory support was maintained for up to three days (minimum, 0 h; maximum, 72 h; mean, 7.212 h; SD ± 15.392 h). Six patients (12.8%) were administered adrenaline or noradrenaline; nine (19.1%) received other medications for cardiovascular support, including dobutamine. Antiseizure medication was administered in five cases (10.6%), while three patients (6.4%) showed seizures during hospitalization. Eight children (17.0%) showed pathological electroencephalography (EEG) results; five children (10.6%) developed brain edema. Antibiotics came into use in 29 patients (61.7%), in most cases, Ampicillin/Sulbactam. Other medications applied included analgesics, proton pump inhibitors (PPIs), bronchospasmolytics, corticosteroids, mannitol, and expectorants. A quarter of the patients (*n* = 11 (23.4%)) showed signs of proteinuria; kidney failure was observed in four (8.5%). None developed liver failure; coagulopathy was seen in one child (2.1%). Almost a third of the patients (*n* = 14 (29.8%)) experienced acute respiratory distress syndrome (ARDS) during hospitalization.

### 3.6. Outcome and Prognostic Variables

In total, 39 patients (83.0%) were discharged with no resulting morbidity. Seven children had a bad outcome, of which four showed some form of neurological impairment (8.5%), and three died in the hospital (6.4%).

Two patients (4.3%) left with an impairment of consciousness; speech impairment was present in three (6.4%); sensory disorders were present in three (6.4%); and increased epileptic potentials were present in one case (2.1%), and several diagnoses per patient were possible. Long-term consequences at the time of readmission were sleep-related apnea, speech development disorders, and one case of left-dominant spastic-dyskinetic tetraparesis. Twelve children (21.3%) were re-admitted to LDP, which was not limited to follow-up examinations (see Figure 2). 

Based on previous findings [9,10,19], we compiled prognostic parameters and related them to our outcome groups. An overview of the relevant parameters can be obtained from Table 3. 

Discharge without resulting morbidity was significantly more probable at a GCS above 3 points (relative risk (RR), 3.333; 95% confidence interval (95%CI), 1.393–8.591), and the absence of cardiovascular arrest improved the chances of a good outcome (RR, 1.389; 95%CI, 1.088–1.773). RR was not found to be significant for a submersion time < 5 min (RR, 1.500; 95%CI, 0.838–2.509); in children with blood glucose levels < 15 mmol/L (RR, 5.625; 95%CI, 0.938–33.739); in children showing pupillary light response (RR, 3.7; 95%CI, 0.676–20.247); in children with a body temperature > 32 °C (RR, 1.595; 95%CI, 0.833–3.056); and when there was no ARDS (RR, 1.458; 95%CI, 0.977–2.177). 

Blood pH values < 7 significantly increased the risk of neurological impairment or death by more than tenfold (RR, 12.3; 95%CI, 4.169–36.49); similarly, a base excess (BE) < −15 mmol/L (RR, 34.000; 95%CI, 4.930–234.460) and blood lactate > 14 mmol/L increased the risk of a bad outcome by a factor of nine (RR, 9.000; 95%CI, 3.573–22.673).

## 4. Discussion

This retrospective study aims to resume research in Leipzig [11] in order to seek trends throughout the last decade and validate earlier data [10,39]. Drowning in children still appears to be a neglected field in research [1]. Relatives and caregivers often see themselves challenged by children’s disabilities and financial burdens [5]. 

Our study included 47 children admitted to Leipzig University Department of Pediatrics (LDP) due to fatal and nonfatal drowning incidents over a period of twelve years. LDP is the city’s biggest pediatric healthcare provider and plays an important role due to its status as a university hospital in both research and patient care. The small case number exposes the infrequency with which even university medical staff in this area deal with the treatment of drowning victims.

In 2021, 299 people died due to drowning in the whole of Germany, of which 47 were 0–20 years old [2]. The estimated number of nonfatal drownings exceeds this by multiple times [4,11]. Overall, from 1994 to 2008, about three drowning incidents per annum were treated at LPD; this number remained stable throughout the following years, 2008–2020.

Patient risk profiles did not change significantly. More than half the children in our case study were between one and three years old, resembling Brüning’s results, and stressed the predominant age risk group [4,11,14,45,46]. Male sex was in the majority, with three-quarters of the patients treated. This reflects recent data from Germany and other countries [2,14,42,47]. Pre-existing health conditions were present in six children (12.8%). This was unlike Brüning’s results but matches the current literature [10,14] and draws attention to the special supervision needs of this patient group. None of our drowning cases happened under the influence of alcohol or other intoxicants, which stands in contrast to data from the US and Sweden where up to 50% of incidents in adolescents involved alcohol or drugs [1,4].

Accident characteristics have remained similar since 2008. Submersion time was estimated from a few seconds to over an hour. Like in the years before, most of the Leipzig incidents took place in private ponds and pools. This differs from what was found by other authors [2,10], where public pools, lakes, and rivers were the main accident locations. Private pools make up the leading incident sites in fatal and nonfatal drownings reported in young children in the United States (US), Spain, and Australia [4,14,45,48], which is similar to our findings. Legislation in the US dictates specific security measurements at pool sites, as this significantly reduces drowning incidents [1,49]. Nowadays, German homeowners are obligated to secure their properties to prevent accidents in hazardous places [50]. As repeatedly presented in this study, more specific requirements appear to be appropriate to effectively assist in the safeguarding of pools and ponds. These include fences, pool alarms, covering, waterfront surveillance, and accessible flotation devices [1,4,14,51]. An increase in the total number of drowning accidents happening in private pools and ponds has not been seen since 2008. In most cases, children were unattended by their parents when the accident happened. Reference can be drawn to Brüning’s data and other authors’ results [11,14,45]. With most drownings happening in the afternoon between 2 pm and 6 pm, this, once more, was the critical time slot identified. This emphasizes the importance of sufficient supervision through caregivers and adequate security measures when preventing drowning in children [4,10,11]. As suggested by Saluja et al., supervision is not to be characterized as the mere presence or absence of caregivers but as a three-dimensional concept: “attention, proximity and continuity” [28], e.g., by always keeping children as close as touchable or no more than an arm’s length away [25]. A peak of cases was again observed during the summer months and weekends, presumably as a result of increased outdoor activity. Similar results were observed by Shaikh in 2016 [42]. Unlike previous years, no second accumulation during winter was confirmed in this follow-up, possibly related to climatic changes preventing the freezing of lakes [52].

In terms of primary care and rescue measurements, circumstances have improved. According to current directives, 95% of emergencies in the federal state of Saxony must be reached within twelve minutes [53]. From 1994 to 2008, 45.5% of cases met that goal, and from 2008 to 2020, this ratio increased to 74%. The average arrival time decreased from 15.7 min to just under 10, successfully achieving the required time frame. Similar results were observed in a recent study from Galicia, Spain [45], where similar emergency medical services (EMS) response times were accomplished. They were assumed to have contributed to high survival. Quan et al. (2016) also found a correlation between shorter EMS response times and good outcomes [41]. The use of pre-clinical medication has declined since 2008. In total, 40.9% of children in the first study received catecholamines, whereas, in our cases, 17% of children were administered adrenaline or noradrenaline, and 15% were administered dobutamine or atropine prior to hospitalization. Further, 38.6% of patients up to 2008 had to be resuscitated by the emergency physician compared with 14.9% up to 2020. The median resuscitation duration remained about the same (39 vs. 45 min (2010)). Pre-clinical intubation was necessary in half (47.7%) of Brüning’s cases and has since declined to a quarter (25.5%). Presumably, differences can be explained by shorter EMS response times [54]. 

Hospitalization duration shortened within the last decade. In the previous cases, the average length of hospital stays amounted to 7.5 days (minimum, 0 d; maximum, 85; median 3 d). From 2008 to 2020, the average patient stayed in the PICU for 3.5 days and in the pediatric ward for 1.5 days.

Concerning neurological outcomes, the portion of patients with a bad outcome has decreased. In the first report, five patients showed neurologic sequelae (11.4%), and twelve children died (25.5%). From 2008 to 2020, the percentage of children with neurological deficits after drowning rescues amounted to 8.5%, which is less than before and congruent with the recent literature. Brüning noted a lethality of 27%; other authors refer to about 10–35% [10,41,45]. At 6.5%, lethality was notably smaller in our cohort. Following a study from the US, there were 13 survivors per drowning death [4]. We counted 15 surviving children per drowning-related death. The proportion of children surviving nonfatal drowning incidents with no signs of neurological impairment was 61.4% in Leipzig up to 2008. Meanwhile, it has been reported to be at around 80% [10,45], which we can prove with our data set (83%). This suggests a general trend toward better outcomes following nonfatal drowning.

Previously suggested prognostic parameters [10,11,39,55] have been adjusted and statistically proven to significantly determine patients’ chances of survival without resulting morbidity. They are summarized in Table 4. We did not observe, as suggested by others [10,43,47], a significant correlation of worse outcomes with the male sex.

In drowning accidents, the length of the hypoxic condition is crucial, especially to the brain [11], explaining the correlation between submersion times and patients’ overall outcomes [6,56]. Submersion times exceeding ten minutes make it highly unlikely to accomplish good outcomes [6,18,41]. As cited by Travers et al., some authors suggest that a submersion time above 25 min is a predictor of death following drowning accidents, while submersion below 15–25 min could still imply survival [40]. Among our cases, there was only one instance of a submersion time of <15–25 min with the patient surviving without resulting morbidity, and we observed one instance of submersion >25 min, with the patient experiencing death from drowning. Limited to this small number of cases, we did not consider a direct comparison insightful.

Reduced blood pH suggests hypoxic acidosis [57], indicating prolonged cerebral hypoxic periods. In our cases, neuron-specific enolase (NSE) was constantly elevated, suggesting effects on the central nervous system. Thus, NSE in itself does not seem an eligible prognostic parameter. The fast removal of drowning victims from the water and sufficient oxygen supplies appear to be vital to improving chances of a good outcome [10,11]. A recent study on pediatric drowning incidents in Australia revealed that 45% of children did not receive cardiopulmonary resuscitation (CPR). Given the fact that only one of the patients in our cohort was not resuscitated by non-medical bystanders prior to the arrival of the emergency physician, we can deduce sufficient caregiver knowledge. Performing CPR remains the only way to prevent drowning victims with cardiac arrest from dying, so further education of the population must remain a priority [1,48].

In the course of drowning, conscious breath holding, diving responses, and fear of drowning result in autonomic conflicts that cause cardiac dysrhythmias, possibly followed by asystole [6,7,9]. A US study found that, upon admission, patients’ hemodynamic statuses in the PICU were very predictive of outcome, even more so than their neurological statuses [46]. Of the children treated from 1994 to 2008, a quarter survived when admitted to the hospital in a life-threatening state. By 2020, 72% of patients who experienced cardiac arrest at any point in time following a nonfatal drowning accident survived without neurological impairment. These data were difficult to compare, as there is no uniform definition of a “life-threatening state” that was applicable to our patient group. As observed before [11], a stable circulatory system upon hospital admission did not necessarily lead to a good outcome. However, we were able to prove the absence of cardiovascular arrest in a patient as a significant predictor for a discharge without resulting morbidity. Moreover, in our cohort, GCS results did not include as many peak or minimal values as described by Brüning et al. There were fewer patients with a GCS of 3 points (21.3% vs. 36.4%) but also fewer with a GCS of 15 points (27.6% vs. 40.9%). Cerebral edema was observed in 20% of patients up to 2008, and this was halved up to 2020 (10.6%). In both Brüning’s findings and our findings, two-thirds of the children (65%) were admitted in a stable circulatory state; a third (32–36%) were hypothermic. Drowning victims often experience hypothermia as a consequence of water temperatures below thermoneutrality [10]. While a body temperature < 28 °C may result in cardiac arrhythmia and cardiac arrest, it also reduces metabolism activity, augmenting acidosis and disrupting coagulatory function [4,6,7]. The significance of a previously considered protective hypothermic body temperature [6,12] was not shown in this study. This is congruent with analyses by Quan et al., who found no correlation between water temperature and patients’ neurological outcomes [18,41]. There was also no significant advantage to therapeutic hypothermia versus normothermia in children with cardiac arrest after drowning accidents [58].

Following drowning, the formation of pulmonary atelectasis may cause pulmonary edema, pneumonia, and ARDS [10,11]. From 1994 to 2008, breathing upon admission was physiological in 39%. In our cases, 53% showed spontaneous respiration. Cumulatively, pneumonia and ARDS were documented as drowning complications in 18% of cases by Brüning. Our results (ARDS in 29.8%) correspond more to data from Raess et al., where a pathological chest X-ray was recorded in 41.3% [10] and was found to be predictive of the overall outcome. Cohen et al. found in 2019 that respiratory distress and lung crepitations are to be considered parameters for the admission of non- or mildly symptomatic children following drowning [59]. 

Structurally, there has been a pleasing development since the results published by Brüning. While in 2008 3 patients (6.3%) had to be excluded from research due to missing medical records, we were able to include all 47 patients. Only one patient’s overall outcome could not be obtained from the records, as there was no final physician’s letter. Emergency protocols were present in 100% of our eligible patients (vs. 80% in 2008); however, they were not always filled in sufficiently. Detailed documentation should be further prioritized to facilitate good clinical practice.

We are aware of several limitations to this study. Firstly, the retrospective study design confines explorations to existing data. The 2015 Utstein-style recommended guidelines [16] were not applied or only partially applied. The process of clinical decision making was not always documented. This impeded the evaluation of confounding variables and limited possible conclusions. A lot of information was lost due to inconsistent documentation [45]. Time indications were often estimations rather than exact information, e.g., concerning reported submersion times that were never precisely measured, as they occurred in the course of accidents. 

Secondly, our data regarded all children from ages 0 to 18 and compared the clinical and paraclinical parameters of various age groups that could possibly differ from one another. More precise differentiation seems appropriate in further study designs. 

Thirdly, the children’s long-term outcomes could not be monitored as there was no regular reappointment with the discharged patients. Therefore, systematic follow-up examinations over the lifespans of these patient groups are strongly recommended.

Moreover, due to the small size of the patient cohort and outcome groups, significance was hard to prove. For some outcome parameters, trends can be assumed and might be affirmed with a bigger patient cohort. Due to the clinic’s status as a university hospital, possible bias can be assumed in both ways: either the cases are more severe than average, or there are more cases resulting from the larger catchment area. Prospective multicenter studies remain necessary to enable sensible conclusions.

According to the World Health Organization (WHO), even in high-income countries, both fatal and nonfatal drowning are underreported, consequently leading to an international undervaluation of the full extent of the burden of drowning incidents [60]. We highly recommend including a broader patient spectrum than just inpatients in subsequent analyses. As deduced from clinical experience, a minor proportion of children is already discharged at the scene, most likely manifesting good outcomes, and a substantial proportion might be from the outpatient department. These cases are missing in this report, although they should be considered when thoroughly examining strategies for the prevention and management of drowning accidents in children.

Lastly, there is a possible bias in our approach to analyzing the data. As this was intended as a follow-up study, we were orientated toward the investigation of preliminary work.

## 5. Conclusions

In this one-center–same-region follow-up, drowning risk factors concerning age, sex, and incident characteristics were confirmed. Special supervision needs apply to male children aged 1–3 years and children with pre-existing health conditions around private ponds and pools during the summer and weekends. Mean hospitalization duration shortened, and morbidity and lethality decreased in cases treated in Leipzig since 2008. There was structural improvement in terms of primary care and medical documentation. 

Parameters suggesting a good outcome after a nonfatal drowning incident include submersion time < 5 min, GCS 4–15, spontaneous movement upon admission, remaining pupillary light response, the absence of cardiovascular arrest, body temperature > 32 °C, pH > 7, blood glucose < 15 mmol/L, lactate < 14 mmol/L, BE ≥ −15 mmol/L, and the absence of ARDS.

Assuming that drowning incidents, both fatal and nonfatal, are still an understudied public health issue, foremost in low- and middle-income countries, data from Germany can only present a very small proportion of the full scale. Even though morbidity and lethality were observed to have decreased within the last decade, we found that epidemiology and risk factors have not changed significantly. Moreso, there are evidence-based measures that should finally be applied to prevention.

We conclude that clear legislation can contribute to safeguarding hazardous places by finally specifying unambiguous measures, like the rule for pool fencing for German pool owners that has not been put into place yet. As most incidents were observed in private home environments, prevention should focus on the low-threshold education of families, extending to as far as, e.g., expectant grandparents. A suitable suggestion would be compulsory participation in basic water competency training for children and their caregivers. Especially with young children, parents and any involved caregivers should be clearly taught to apply situation-adequate ways of keeping vigilance over those under protection when around bodies of water, e.g., by never moving more than an arm’s length away. Recalling that performing CPR remains the only way to prevent drowning victims with cardiac arrest from dying, the ongoing education and practical instruction of the whole population about resuscitation skills must be prioritized as a social obligation.

We further conclude that profound scientific documentation of drowning accidents should be facilitated by supplying physicians and emergency personnel with uniform record forms oriented toward internationally adopted rationales. Specifically addressed emergency and study protocols should incorporate Utstein-style recommended guidelines. 

Subsequent investigations should consider prospective randomized-controlled multi-center studies with bigger patient cohorts and thereby include affected children already discharged at the scene or sent home directly from the emergency department—rather than just inpatients—in order to gain full insights. Further, systematic follow-up examinations over the lifespans of nonfatal drowning patients are highly recommended. 

Regular status investigations of consistent regions could be useful for process evaluations of drowning epidemiology. We see particular importance in standardized procedures and documentation to enable worldwide data comparability.

## Figures and Tables

**Table 1:** Laboratory testing results at time of admission (47 children).

Parameter	Reference	Minimum	Maximum	Median	Mean	SD
pH	7.35–7.45	6.567	7.450	7.305	7.243	0.219
Base Excess (mmol/L)	0.0 ± 2.0	1.5	−32.1	−5.5	−7.832	8.718
pCO_2_ (mmHg)	35–48	14.9	82.0	41.95	44.158	13.008
SpO_2_ (%)	95–99	75	100	96	93.595	6.890
Lactate (mmol/L)	0.50–1.60	0.80	22.00	3.60	5.187	4.754
NSE (µg/L)	1.38–22.08	28.90	83.44	49.40	51.571	14.284
Leucocyte Count (10^9^/L)	6.5–15	3.4	30.6	11.8	12.132	5.399
Blood Glucose (mmol/L)	3.89–5.82	4.70	24.60	7.60	9.197	4.951

**Table 2:** Selected clinical parameters upon hospital admission (47 children).

Parameter	Minimum	Maximum	Median	Mean	SD
Body temperature (°C)	27.0	38.4	36.5	43.973	3.079
Systolic blood pressure (mmHg)	45	160	110	110.343	20.253
Diastolic blood pressure (mmHg)	20	105	60	63.314	17.008
Heart rate (bpm)	65	150	112	113.262	21.112
**Pupillary status**	**Round, equal**	**Distorted**			
*n* (%)	45 (95.7)	2 (4.3)			
**Pupillary light response**	**Prompt**	**Delayed**	**None**		
*n* (%)	38 (80.9)	3 (6.4)	4 (8.5)		
**GCS (points)**	**15–14**	**13–12**	**11–9**	**8–3**	**N/A ^1^**
*n* (%)	14 (29.8)	2 (4.3)	4 (8.5)	14 (29.8)	13 (27.6)

^1^ Not applicable.

**Table 3:** Prognostic parameters for patients’ overall outcomes (46 children).

	Parameter at Time of Admission	Total		No Resulting Morbidity		Neurological Impairment or Death		Significance ^2^	
		*n* = 46 ^1^		*n* = 39		*n* = 7			
		*n*	(%)	*n*	(%)	*n*	(%)	*p*	z
	**Sex**							0.521	−0.641
1	male	35	(76.1)	29	(82.9)	6	(17.1)		
2	female	11	(23.9)	10	(90.9)	1	(9.1)		
	N/A ^3^	0	(0.0)	-	-	-	-		
	**Submersion time**							0.049	−1.968
1	<5 min	32	(69.6)	29	(90.6)	3	(9.4)		
2	≥5 min	8	(17.4)	5	(62.5)	3	(37.5)		
	N/A ^3^	6	(13.0)	-	-	-	-		
	**GCS**							<0.001	−4.451
1	3	10	(21.7)	3	(30.0)	7	(70.0)		
2	4–15	23	(50.0)	23	(100.0)	0	(0.0)		
	N/A ^3^	13	(28.3)	-	-	-	-		
	**Spontaneous movement**							<0.001	−5.069
1	yes	35	(76.1)	35	(100.0)	0	(0.0)		
2	no	11	(23.9)	4	(36.4)	7	(63.6)		
	N/A ^3^	0	(0.0)	-	-	-	-		
	**Pupillary light response**							<0.001	−3.708
1	yes	40	(87.0)	37	(92.5)	3	(7.5)		
2	omitting	4	(8.7)	1	(25.0)	3	(75.0)		
	N/A ^3^	2	(4.3)	-	-	-	-		
	**Cardiovascular arrest**							0.009	−2.605
1	no	21	(45.7)	21	(100.0)	0	(0.0)		
2	yes	25	(54.3)	18	(72.0)	7	(28.0)		
	N/A ^3^	0	(0.0)	-	-	-	-		
	**Child’s body temperature**							0.022	−2.291
1	32.0–38.4 °C	34	(73.9)	31	(91.2)	3	(8.8)		
2	<32 °C	7	(15.2)	4	(57.1)	3	(42.9)		
	N/A ^3^	5	(10.9)	-	-	-	-		
	**Blood pH**							<0.001	−4.583
1	<7 mmol/L	4	(8.7)	0	(0.0)	4	(100.0)		
2	≥7 mmol/L	37	(80.4)	34	(91.9)	3	(8.1)		
	N/A ^3^	5	(10.9)	-	-	-	-		
	**Blood glucose**							<0.001	−4.410
1	<15 mmol/L	32	(69.6)	30	(93.8)	2	(6.3)		
2	≥15 mmol/L	6	(13.0)	1	(16.7)	5	(83.3)		
	N/A ^3^	8	(17.4)	-	-	-	-		
	**Lactate**							<0.001	−3.805
1	<14 mmol/L	36	(78.3)	32	(88.9)	4	(11.1)		
2	≥14 mmol/L	3	(6.5)	0	(0.0)	3	(100.0)		
	N/A ^3^	7	(15.2)	-	-	-	-		
	**Base excess**							<0.001	−5.696
1	<−15 mmol/L	6	(13.0)	0	(0.0)	6	(100.0)		
2	≥−15 mmol/L	34	(73.9)	33	(97.1)	1	(2.9)		
	N/A ^3^	6	(13.0)	-	-	-	-		
	**Development of ARDS**							0.011	−2.532
1	no	32	(69.6)	30	(93.8)	2	(6.3)		
2	yes	14	(30.4)	9	(64.3)	5	(35.7)		
	N/A ^3^	0	(0.0)	-	-	-	-		

^1^ One patient’s overall outcome could not be obtained (*n* = 1). ^2^ Mann–Whitney test. ^3^ Not applicable.

**Table 4:** Predicting outcomes after a drowning incident in children.

Parameters Suggesting an Outcome without Resulting Morbidity after a Drowning Incident in Children
⇒ Submersion time below 5 min
⇒ GCS of 4–15 points
⇒ Spontaneous movement upon admission
⇒ Remaining pupillary light response
⇒ Absence of cardiovascular arrest
⇒ Body temperature above 32 °C
⇒ Blood pH > 7 mmol/L
⇒ Blood glucose < 15 mmol/L
⇒ Lactate level < 14 mmol /L
⇒ Base excess ≥ −15 mmol/L
⇒ Absence of ARDS

## Data Availability

The original data presented in this study are included in the Supplementary Material; further inquiries can be directed to the corresponding authors.

## Supplementary Materials

The following supporting information can be downloaded at: https://www.mdpi.com/article/10.3390/children11040439/s1, Table S1: RAW_DATA_FILE_ DROWNING.xlsx.

## References

[1] World Health Organization (2014). Global Report on Drowning: Preventing a Leading Killer.

[2] DLRG Bundesverband Todesfälle Nach Altersgruppen. https://www.dlrg.de/informieren/die-dlrg/presse/statistik-ertrinken/.

[3] Bundesarbeitsgemeinschaft Mehr Sicherheit Für Kinder e. V. Häufigste Unfallarten Mit Todesfolge. https://www.kindersicherheit.de/fachinformationen/unfallstatistiken.html.

[4] Layon A.J., Modell J.H., Warner D.S., Warner M.A. (2009). Drowning: Update 2009. Anesthesiology.

[5] Peden M. (2008). World Health Organization World Report on Child Injury Prevention.

[6] Szpilman D., Bierens J.J.L.M., Handley A.J., Orlowski J.P. (2012). Drowning. N. Engl. J. Med..

[7] Bierens J.J.L.M., Lunetta P., Tipton M., Warner D.S. (2016). Physiology of Drowning: A Review. Physiology.

[8] Orlowski J.P., Szpilman D. (2001). DROWNING: Rescue, Resuscitation, and Reanimation. Pediatr. Clin. N. Am..

[9] Szpilman D., Sempsrott J., Webber J., Hawkins S.C., Barcala-Furelos R., Schmidt A., Queiroga A.C. (2018). ‘Dry Drowning’ and Other Myths. CCJM.

[10] Raess L., Darms A., Meyer-Heim A. (2020). Drowning in Children: Retrospective Analysis of Incident Characteristics, Predicting Parameters, and Long-Term Outcome. Children.

[11] Brüning C., Siekmeyer W., Siekmeyer M., Merkenschlager A., Kiess W. (2010). Retrospektive Analyse von 44 Ertrinkungsunfällen von Kindern und Jugendlichen. Wien. Klin. Wochenschr..

[12] Gries A. (2001). Notfallmanagement bei Beinahe-Ertrinken und akzidenteller Hypothermie. Anaesthesist.

[13] Kluger G.J., Kirsch A., Hessenauer M., Aust H., Berweck S., Sperl W., Betzler C., Stülpnagel-Steinbeis C., von Staudt M. (2019). Unresponsive Wakefulness Syndrome in Children after Near-Drowning: Long-Term Outcome and Impact on the Families. Neuropediatrics.

[14] Chang S.S.M., Ozanne-Smith J. (2020). Drowning Mortality in Children Aged 0–14 Years in Victoria, Australia: Detailed Epidemiological Study 2001–2016. Inj. Prev..

[15] Bratton S.L., Jardine D.S., Morray J.P. (1994). Serial Neurologic Examinations after Near Drowning and Outcome. Arch. Pediatr. Adolesc. Med..

[16] Idris A.H., Bierens J.J.L.M., Perkins G.D., Wenzel V., Nadkarni V., Morley P., Warner D.S., Topjian A., Venema A.M., Branche C.M. (2017). 2015 Revised Utstein-Style Recommended Guidelines for Uniform Reporting of Data from Drowning-Related Resuscitation: An ILCOR Advisory Statement. Resuscitation.

[17] Dean J.M., Kaufman N.D. (1981). Prognostic Indicators in Pediatric Near-Drowning: The Glasgow Coma Scale. Crit. Care Med..

[18] Quan L., Mack C.D., Schiff M.A. (2014). Association of Water Temperature and Submersion Duration and Drowning Outcome. Resuscitation.

[19] Vähätalo R., Lunetta P., Olkkola K.T., Suominen P.K. (2014). Drowning in Children: Utstein Style Reporting and Outcome. Acta Anaesthesiol. Scand..

[20] Girasek D.C., Hargarten S. (2022). Prevention of and Emergency Response to Drowning. N. Engl. J. Med..

[21] Dakessian A., Bachir R., El Sayed M. (2019). Impact of Trauma Designation Levels on Survival of Drowning Victims: An Observational Study from Trauma Centers in the United States. Medicine.

[22] Moreland B., Ortmann N., Clemens T. (2022). Increased Unintentional Drowning Deaths in 2020 by Age, Race/Ethnicity, Sex, and Location, United States. J. Saf. Res..

[23] Franklin R.C., Peden A.E., Hamilton E.B., Bisignano C., Castle C.D., Dingels Z.V., Hay S.I., Liu Z., Mokdad A.H., Roberts N.L.S. (2020). The Burden of Unintentional Drowning: Global, Regional and National Estimates of Mortality from the Global Burden of Disease 2017 Study. Inj. Prev..

[24] Meddings D.R., Scarr J.-P., Larson K., Vaughan J., Krug E.G. (2021). Drowning Prevention: Turning the Tide on a Leading Killer. Lancet Public Health.

[25] Denny S.A., Quan L., Gilchrist J., McCallin T., Shenoi R., Yusuf S., Hoffman B., Weiss J., Agran P.F., Hirsh M. (2019). Prevention of Drowning. Pediatrics.

[26] Spencer M., Hedegaard H., Warner M., National Center for Health Statistics (USA) (2021). Unintentional Drowning Deaths among Children Aged 0–17 Years, United States, 1999–2019.

[27] Driscoll T.R., Harrison J.A., Steenkamp M. (2004). Review of the Role of Alcohol in Drowning Associated with Recreational Aquatic Activity. Inj. Prev..

[28] Saluja G., Brenner R., Morrongiello B.A., Haynie D., Rivera M., Cheng T.L. (2004). The Role of Supervision in Child Injury Risk: Definition, Conceptual and Measurement Issues. Inj. Control. Saf. Promot..

[29] Anderson K.R., Ramos W.D., Schuman J.T. (2021). The Role of Permission, Supervision, and Precipitating Events in Childhood Pool/Spa Submersion Incidents, United States, 2000–2017. Int. J. Environ. Res. Public Health.

[30] De Buck E., Vanhove A.-C., Veys D.O.K., Lang E., Vandekerckhove P. (2021). Day Care as a Strategy for Drowning Prevention in Children under 6 Years of Age in Low- and Middle-income Countries. Cochrane Database Syst. Rev..

[31] Thompson D.C., Rivara F. (1998). Pool Fencing for Preventing Drowning of Children. Cochrane Database Syst. Rev..

[32] Sandomierski M.C., Morrongiello B.A., Colwell S.R. (2019). Near Water: An Intervention Targeting Parent Beliefs About Children’s Water Safety. J. Pediatr. Psychol..

[33] Bierens J., Abelairas-Gomez C., Furelos R.B., Beerman S., Claesson A., Dunne C., Elsenga H.E., Morgan P., Mecrow T., Pereira J.C. (2021). Resuscitation and Emergency Care in Drowning: A Scoping Review. Resuscitation.

[34] Barcala-Furelos R., Graham D., Abelairas-Gómez C., Rodríguez-Núñez A. (2021). Lay-Rescuers in Drowning Incidents: A Scoping Review. Am. J. Emerg. Med..

[35] Szpilman D., Morgan P.J. (2021). Management for the Drowning Patient. Chest.

[36] Bierens J., Bray J., Abelairas-Gomez C., Barcala-Furelos R., Beerman S., Claesson A., Dunne C., Fukuda T., Jayashree M.T., Lagina A. (2023). A Systematic Review of Interventions for Resuscitation Following Drowning. Resusc. Plus.

[37] Koon W., Clemens T., Bierens J., Quan L. (2021). Studying Outcome Predictors of Drowning at the Scene: Why Do We Have so Few Answers?. Am. J. Emerg. Med..

[38] Suominen P.K., Vähätalo R. (2012). Neurologic Long Term Outcome after Drowning in Children. Scand. J. Trauma. Resusc. Emerg. Med..

[39] Pöppelmann M. (2008). Retrospektive Analyse Pädiatrischer Ertrinkungsfälle Und Entwicklung Eines Prognostischen Scores.

[40] Travers A.H., Perkins G.D., Berg R.A., Castren M., Considine J., Escalante R., Gazmuri R.J., Koster R.W., Lim S.H., Nation K.J. (2015). Part 3: Adult Basic Life Support and Automated External Defibrillation. Circulation.

[41] Quan L., Bierens J.J.L.M., Lis R., Rowhani-Rahbar A., Morley P., Perkins G.D. (2016). Predicting Outcome of Drowning at the Scene: A Systematic Review and Meta-Analyses. Resuscitation.

[42] Shaikh M.A. (2012). Epidemiology of Drowning and near Drowning at Karachi Beaches from 2012 to 2014. J. Pak. Med. Assoc..

[43] Graf W.D., Cummings P., Quan L., Brutocao D. (1995). Predicting Outcome in Pediatric Submersion Victims. Ann. Emerg. Med..

[44] Sindall R., Mecrow T., Queiroga A.C., Boyer C., Koon W., Peden A.E. (2022). Drowning Risk and Climate Change: A State-of-the-Art Review. Inj. Prev..

[45] Sánchez-Lloria P., Barcala-Furelos R., Otero-Agra M., Aranda-García S., Cosido-Cobos Ó., Blanco-Prieto J., Muñoz-Barús I., Rodríguez-Núñez A. (2022). Análisis descriptivo de las causas, consecuencias y respuesta de los sistemas de Salud Pública en los ahogamientos pediátricos en Galicia. Un estudio retrospectivo de 17 años. Rev. Esp. Salud Pública.

[46] Habib D.M., Tecklenburg F.W., Webb S.A., Anas N.G., Perkin R.M. (1996). Prediction of Childhood Drowning and Near-Drowning Morbidity and Mortality. Pediatr. Emerg. Care.

[47] Peden A.E., Işın A. (2022). Drowning in the Eastern Mediterranean Region: A Systematic Literature Review of the Epidemiology, Risk Factors and Strategies for Prevention. BMC Public Health.

[48] Awan B., Wicks S., Peden A.E. (2022). A Qualitative Examination of Causal Factors and Parent/Caregiver Experiences of Non-Fatal Drowning-Related Hospitalisations of Children Aged 0–16 Years. PLoS ONE.

[49] Rimsza M.E., Schackner R.A., Bowen K.A., Marshall W. (2002). Can Child Deaths Be Prevented? The Arizona Child Fatality Review Program Experience. Pediatrics.

[50] (2002). § 823. Bürgerliches Gesetzbuch in der Fassung der Bekanntmachung.

[51] Blazovic S., Jamal Z., Quinn K. (2022). Pool Safety.

[52] DWD Climate Data Center (CDC): Annual Regional Averages of Air Temperature (Annual Mean) in °C (2 m above Ground) for Germany, Version V19.3. https://opendata.dwd.de/climate_environment/CDC/regional_averages_DE/annual/air_temperature_mean/DESCRIPTION_regional_averages_DE_annual_air_temperature_mean_en.pdf.

[53] Sächsischer Landesrettungsdienstplan Vom 30. November 1994 (SächsABl. S. 1526), Enthalten in Der Verwaltungsvorschrift Vom 14. Dezember 2005 (SächsABl. SDr. S. S 758). https://www.revosax.sachsen.de/vorschrift/2271-Saechsischer-Landesrettungsdienstplan.

[54] Holmén J., Herlitz J., Ricksten S., Strömsöe A., Hagberg E., Axelsson C., Rawshani A. (2020). Shortening Ambulance Response Time Increases Survival in Out-of-Hospital Cardiac Arrest. J. Am. Heart Assoc..

[55] Plubrukarn R., Tamsamran S. (2003). Predicting Outcome in Pediatric Near-Drowning. J. Med. Assoc. Thai.

[56] Pearn J. (1985). The Management of near Drowning. BMJ.

[57] Oehmichen M., Hennig R., Meissner C. (2008). Near-Drowning and Clinical Laboratory Changes. Leg. Med..

[58] Moler F.W., Hutchison J.S., Nadkarni V.M., Silverstein F.S., Meert K.L., Holubkov R., Page K., Slomine B.S., Christensen J.R., Dean J.M. (2016). Targeted Temperature Management after Pediatric Cardiac Arrest Due to Drowning: Outcomes and Complications. Pediatr. Crit. Care Med..

[59] Cohen N., Capua T., Lahat S., Glatstein M., Sadot E., Rimon A. (2019). Predictors for Hospital Admission of Asymptomatic to Moderately Symptomatic Children after Drowning. Eur. J. Pediatr..

[60] World Health Organization (2023). Hidden Depths: The Global Investment Case for Drowning Prevention.

